# Does lower educational attainment increase the risk of osteoarthritis surgery? a Swedish twin study

**DOI:** 10.1186/s12891-023-06163-w

**Published:** 2023-01-28

**Authors:** Maria Lindéus, Aleksandra Turkiewicz, Karin Magnusson, Martin Englund, Ali Kiadaliri

**Affiliations:** 1 Faculty of Medicine, Department of Clinical Sciences Lund, Clinical Epidemiology Unit, Lund University, OrthopaedicsLund, Sweden; 2 Norwegian Institute of Public Health, Cluster for Health Services Research, Oslo, Norway; 3 Centre for Economic Demography, Lund University, Lund, Sweden

**Keywords:** Twin study, Osteoarthritis surgery, Inequalities, Education

## Abstract

**Background:**

Previous studies have reported an inverse association between educational attainment and different osteoarthritis (OA) outcomes. However, none of the previous studies have accounted for potential confounding by early-life environment and genetics. Thus, we aimed to examine the association between educational attainment and knee and hip OA surgery using twin data.

**Methods:**

From the Swedish Twin Registry (STR), we identified dizygotic (DZ) and monozygotic (MZ) twins. All twins in the STR aged 35 to 64 years were followed from January the 1^st^ 1987 or the date they turned 35 years until OA surgery, relocation outside Sweden, death or the end of 2016 (18,784 DZ and 8,657 MZ complete twin pairs). Associations between educational attainment and knee and hip OA surgery were estimated in models matched on twin pairs, using Weibull within-between (WB) shared frailty model.

**Results:**

For knee OA surgery, the analysis matched on MZ twins yielded a within-estimate hazard ratio (HR) per 3 years of education, of 1.06 (95% CI: 0.81, 1.32), suggesting no association between the outcome and the individual´s education. Rather, there seemed to be a so called familial effect of education, with a between-pair estimate of HR = 0.71 (95% CI: 0.41, 1.01). For hip OA surgery, the within- and between-pair estimates for MZ twins were 0.92 (95% CI: 0.69, 1.14) and 1.15 (95% CI: 0.87, 1.42), respectively.

**Conclusions:**

Our results suggest that the inverse associations between education and knee/hip OA surgery observed in cohort studies are potentially confounded by unobserved familial factors like genetics and/or early life exposures.

**Supplementary Information:**

The online version contains supplementary material available at 10.1186/s12891-023-06163-w.

## Background

Osteoarthritis (OA) is a major cause of disability in the Nordic region [1] and globally [2], being the 18^th^ leading cause of global years lived with disability in people aged 50–74 years in 2019 [2]. There exists no disease-modifying treatment for OA, i.e. the treatment is focused towards reducing symptoms. Around 30% of knee and 14% of hip OA patients have total joint replacement surgery when OA symptoms are not alleviated by other treatments [3]. About 30,000 knee and hip surgeries were performed due to OA in Sweden in 2019 [4, 5].

Although the aetiology of OA is not fully understood, known causes include both genetic and environmental factors. Swedish, Norwegian and Danish twin register studies have reported that genetics contribute to 18%-53% and 47–73% of the variance in knee and hip OA surgery, respectively [6–8]. Accordingly, a substantial part of the variance in OA surgery is due to environmental factors.

One important environmental factor with large impact on health is socioeconomic status (SES) [9], which is largely determined by educational level. Previous studies suggest that having higher education will decrease a person’s risk of OA/more symptomatic OA [10–17]. Further, a Swedish study of the association between educational level and knee and hip OA surgery reported that the rate of knee arthroplasty due to OA was higher in people with lower educational attainment, whereas results concerning hip OA were less clear [18]. Several studies have examined the association between OA surgery and SES using other proxies than education, but these proxies have been area-based such as the average household income in the patient’s neighbourhood etc. [19–22].

A major challenge with the reported studies is the lack of adjustment for potentially confounding factors like genetics and early-life exposures. To address this, we propose using a twin design, where twins are treated as pairs matched on familial confounders like genetics (to varying degree in mono- and di-zygotic twins) and early-life exposures such as parental characteristics. If twins with similar genes and early-life exposures are different in terms of education level, any difference in OA surgery outcomes cannot be attributed to genetics or shared early-life exposures. Rather, any observed difference in OA surgery must be due to individual choices or possibilities to reach a certain education level, or individual factors that impact on both the education level and OA surgery. We aimed to examine the association between educational attainment and OA-related surgery. Further, we aimed to investigate to what extent the shared familial factors confound the associations between educational attainment and knee and hip OA surgery using the Swedish Twin Registry (STR), the largest twin cohort in the world.

## Methods

### Data sources

The STR is managed by the Karolinska Institute and contains information about approximately 85,000 Sweden-born twin pairs for which zygosity is known (https://ki.se/en/research/the-swedish-twin-registry). From STR we identified incomplete and complete dizygotic (DZ) and monozygotic (MZ) twin pairs, born between 1922 and 1980, and retrieved information on sex, zygosity, and vital status of the twins. From the Longitudinal integration database for health insurance and labour market studies (LISA) we collected data on each twin’s educational attainment from the year the twin was included in the study. Since LISA only has data on educational attainment from 1990, we used data on education from 1990 for those twins that were included during 1987 to 1989. The LISA is maintained by Statistics Sweden and integrates data from educational- and social sectors and the labour market (https://www.scb.se/lisa-en). We retrieved information about knee and hip OA diagnosis and OA surgery (surgical codes and date of surgery) from 1987 to 2016 from the Swedish National Patient Register, which is maintained by the Swedish National Board of Health and Welfare. Information about each twin’s place of residence at age 18 and migration status was retrieved from the Statistics Sweden’s Population Register. We collected information about BMI and physical fitness at military conscription at the age of 18 from the Swedish Military Conscription Registry. This data was available for the years 1969 to 1998 for males only, i.e. for males born between 1951 and 1980, a group for which military conscription was mandatory. The physical fitness at military conscription was measured as maximal aerobic workload in Watts via a bicycle ergometer test [23]. The BMI was calculated from weight and height measured by trained personnel at conscription.

### Outcome and follow-up period

Since most OA surgeries are done in people aged ≥ 65 years [24] we excluded people aged ≥ 65 years and included these in a sensitivity analysis (the characteristics of these subjects are shown in supplementary material, Table A1-A3). This was done in order to minimize misclassification of the OA-surgery since people aged ≥ 65 years might already have had OA surgery before 1987. The recruitment period was from the 1^st^ of January 1987 to the 31^st^ of December 2015. Each subject’s follow-up started on the 1^st^ of January 1987 or the date he/she turned 35 years old, whichever happened last. The reason to start follow-up from age 35 years and older was because we expect that the majority of people at this age have attained their highest educational level, also OA is very uncommon before age of 35 [25]. The twins were followed until the date of OA surgery, relocation outside Sweden, death, or the 31^st^ of December 2016, whichever occurred first.

### Osteoarthritis surgery

Knee and hip OA surgery were defined as knee (ICD-9 codes: 715, ICD-10 codes: M17) or hip OA diagnosis (ICD-9 codes: 715, ICD-10 codes: M16) registered at the same time with a knee or hip OA-related surgical procedure (arthroplasty or osteotomy). The surgery codes are based on the National Board of Health and Welfare´s classification of surgical procedures from 1963 to 1996 [26] and 1997 to present [27]. For knee OA surgery following surgery codes were used: 8424, 8010, 8428, 8423, 8426, NGB49, NGB19, NGK59, NGB29, NGB59, NGB53, NGB09, NGB39. Following surgery codes were used for hip OA surgery: 8414, 8010, 8409, 8419, NFB49, NFB29, NFB39, NFB99, NFK59, NFB62. For description of the surgery codes see supplementary material, Table A4.

### Covariates

We considered age, sex, birth cohort, early-life place of residence, and early-life BMI and physical fitness as potential confounders of the association between education level and OA surgery. Following five birth cohorts were chosen: 1922–1934, 1935–1944, 1945–1954, 1955–1964, 1965–1980 to account for changing education patterns over time. In order to take geographical variations in health [28] and education [29] into account we adjusted for early-life place of residence by having a four-level variable which described the place of residence each subject had at the age of 18. The four categories were: residence in the south, middle, and north of Sweden and residence in one of three most densely populated counties of the country (supplementary material, Table A5). Studies have found that obesity early in life relates to less educational attainment [30, 31] and obesity [32] in adulthood. Obesity, is in turn an important risk factor for OA [33–35]. What concerns early-life physical fitness, it has been reported that physical activity/performance in childhood/adolescence is positively associated with educational attainment [36, 37]. The evidence regarding the association between early-life physical fitness and osteoarthritis is sparse. Early-life physical fitness in the form of knee-extensor strength in adolescence has been reported to be positively associated with the risk of incident knee OA by middle age [38]. However, it has also been reported a positive association between early-life physical activity and tibial cartilage volume in adulthood [39]. Since BMI and physical fitness in adolescence were only available for males at military conscription at the age of about 18, we adjusted for these in a sensitivity analysis. Physical fitness, measured by maximal aerobic workload in Watts, was categorized into four groups based on quartiles while we kept BMI as a continuous variable.

### Statistical analysis

We transformed the Statistics Sweden’s education variable into years of education with a range from 7 to 20 years, where 7 years represented the most basic education and 20 years represented PhD education (supplementary material, Table A6). Since length of education can be expected to have threshold effects associated with completing a certain educational level, we also performed analyses with educational level as a binary variable, corresponding to educational attainment lower than post-secondary education (< 13 years of education) and at least post-secondary education (≥ 13 years of education). The results concerning education as a binary variable are presented in the supplementary material. Separate analyses were made for knee and hip OA surgery. The associations between educational attainment and knee and hip OA surgery were assessed by estimating hazard ratios (HR) using Weibull models. The HRs were reported per 3 years of education.

We performed a series of analyses to understand the extent to which the association between education and the outcomes were confounded by shared familial factors. First, we analysed unmatched twin pairs that included both incomplete and complete DZ and MZ twin pairs, treating the data as an unmatched cohort and adjusting for age, sex, birth cohort [1922–1934 1935–1944 1945–1954 1955–1964 1965–1980], and early place of residence. Then, we accounted for the matching by using the gamma-Weibull within-between (WB) shared frailty model [40] using Mundlak specification. WB models are well-established in sibling comparison research and are used to decompose the association between exposure and outcome into a “within-cluster effect” and a “between-cluster effect” [41]. In our study, if we assume no individual level confounders, the within effect in MZ twin pairs represents a direct causal effect of educational attainment on OA surgery, as MZ twins share several potential confounders (100% of their genes [42] and their early-life environment). The “between-cluster effect” in our study quantifies the degree of shared familial confounding in the association between educational attainment and OA surgery [41]. In other words, a “between-cluster effect” different from zero reflects “the presence of shared familial confounding” [41]. For the WB analyses we used the R-code provided by Dahlqwist et.al [43]. Besides using Weibull models, we also did analyses using the Cox model and stratified Cox model (supplementary material). We assumed that the potential difference in the unmatched and matched estimates is due to the impact of shared genetic and early-life environmental factors [41]. We assessed DZ and MZ twin pairs combined, and DZ twin pairs and MZ twin pairs separately. Since MZ twins share 100% of their genes in contrast with DZ twins that share, on average, 50% of their genes [42], the difference between the estimates from MZ twin pairs and DZ twin pairs might signal the potential confounding by genetic factors. We evaluated the proportional hazard assumption using plots of Schoenfeld residuals. We found no evidence of violation of this assumption in most models, except for model examining knee OA surgery in the unmatched cohort. However, the slight non-proportionality had no impact on the overall results of the models. Attained age was used as time-scale in the models. For the subgroup analysis in males born between 1951 and 1980, we adjusted the models for BMI and physical fitness at military conscription. In the subgroup analysis, males with missing data on BMI and physical fitness were excluded (*N* = 4,136). For characteristics of the males included in the subgroup analysis see supplementary material, Table A7-A8. Analyses were performed in R version 3.6.3 and Stata 17.

## Results

After excluding 1,077 people with unknown level of education and 1,396 with missing information about place of residence at age 18, a total of 67,071 twins (53.7% females) were included in the study. The whole study sample consisted of 18,784 DZ and 8,657 MZ complete twin pairs, and 12,189 twins without a co-twin. The mean (SD) age at study entry was 41.7 (8.7) and the mean follow-up was 22.6 years for both knee and hip OA surgery. During the follow-up, we identified 2,017 knee OA surgeries and 2,285 hip OA surgeries (Table 1). For characteristics of twin pairs discordant on educational status see supplementary material, Table A9-A10.

**Table 1:** Descriptive data on educational status of the study population

Whole study sample	DZ and MZ complete and incomplete twin pairs		DZ complete twin pairs		MZ complete twin pairs		
	Years of education, continuous						
Years of education, mean years ± SD	11.2 ± 2.9		11.1 ± 2.9		11.5 ± 2.9		
	Years of education, categorical						
	< 13	≥ 13	< 13	≥ 13	< 13	≥ 13	Missing
Number of people, n	47,753	19,318	27,365	10,203	11,703	5,611	1,077
Sex, Females, % missing, %	53.0 0.0	55.6 0.0	52.0 0.0	53.3 0.0	54.7 0.0	58.4 0.0	5.1 86.7
Age at entry, mean age ± SD	42.9 ± 9.1	38.6 ± 6.5	43.2 ± 9.0	39.2 ± 6.8	42.3 ± 8.9	38.1 ± 6.0	44.0 ± 10.9
Knee OA surgery, n	1,681	336	1,009	216	399	75	10
Hip OA surgery, n	1,800	485	1,065	277	421	135	9
Person-years follow-up, knee OA surgery	1,132,628	383,220	676,037	220,142	271,134	102,707	6,592
Person-years follow-up, hip OA surgery	1,130,901	381,587	675,129	219,273	270,593	102,108	6,588

### Knee OA surgery

In the Weibull regression model the unmatched analysis of the total sample yielded a hazard ratio (HR) of 0.90 (95% CI: 0.86, 0.95) for knee OA surgery, indicating that for every three additional years of education the rate of knee OA surgery lowers by ~ 10%. Adjusting for familial confounding through twin design resulted in an HR of 1.05 (95% CI: 0.95, 1.16) suggesting potential familial confounding which is also supported by the “between-effect estimates”. Comparing HRs from DZ and MZ twins suggests that association between education and knee OA surgery is mainly confounded by genetic factors. The estimate in MZ twins was 1.06, 95% CI: 0.81, 1.32, suggesting no association between the individual´s education and the risk of knee OA surgery. Regarding the between-pair estimates, the HR for MZ twins was 0.71 (95% CI: 0.41, 1.01). The result indicates that there exists familiar confounding on the association between educational attainment and knee OA surgery. The results when people aged ≥ 65 years were included were similar (supplementary material, figure A1).

**Fig. 1:**
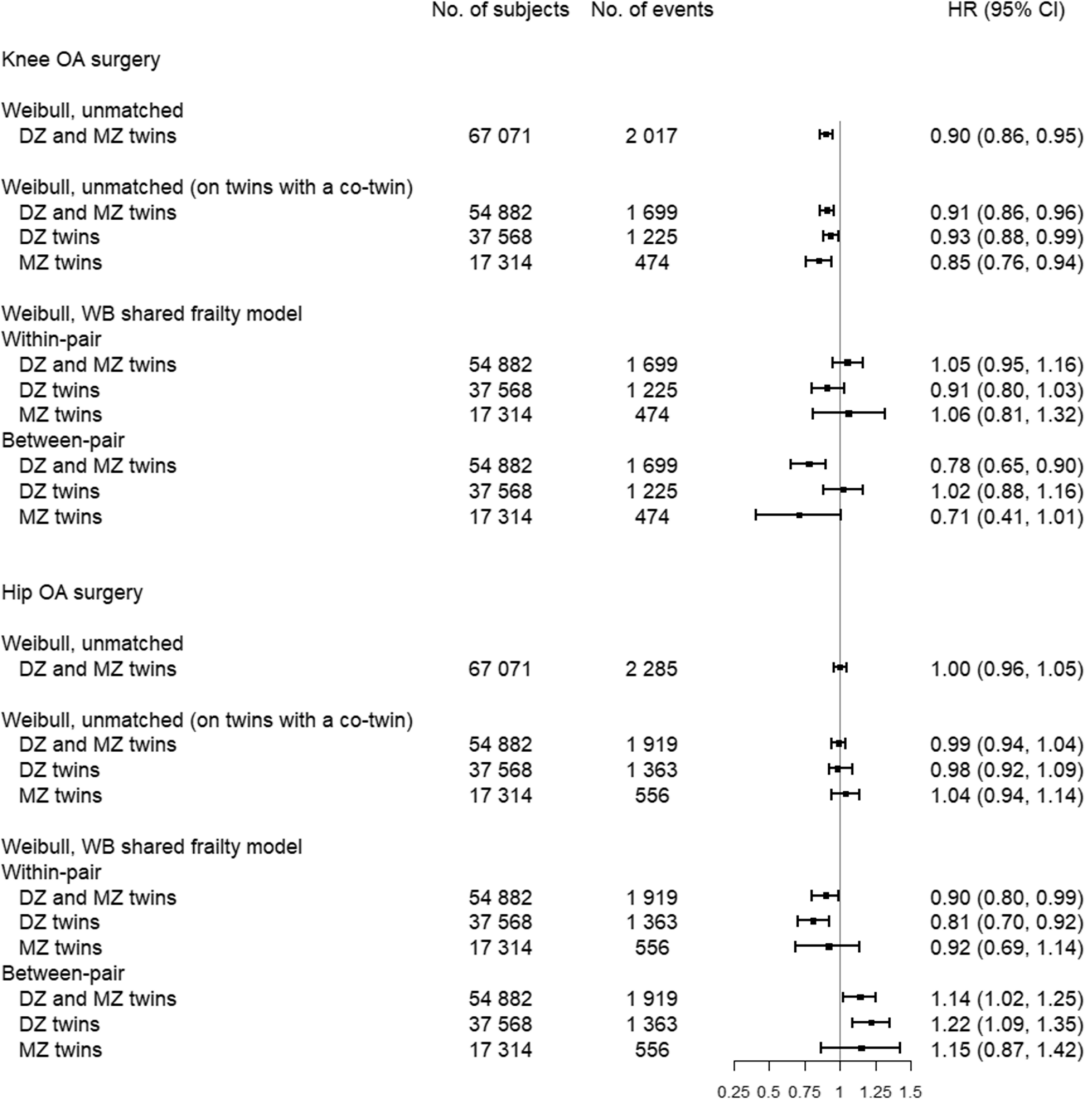
The hazard ratios (HR) per 3 years of education with 95% confidence intervals (CI) for knee and hip OA surgery, for unmatched and matched dizygotic (DZ) and monozygotic (MZ) twins. Models were adjusted for sex, birth cohort, and place of residence at age 18

When treating education as a binary variable, the estimates suggested neither an association between individual educational attainment and the outcome nor any familial confounding on the association (supplementary material, Figure A2-A3). Estimates generated by the Cox and the stratified Cox model, suggested a protective effect of education on the association when education was treated as a binary variable but not as a continuous variable (supplementary material, Figure A4-A5).

### Hip OA surgery

For hip OA surgery, the HR in the unmatched analysis for the total sample was 1.00 (95% CI: 0.96, 1.05). After accounting for matching, the estimate was 0.90 (95% CI: 0.80, 0.99) for total sample, 0.81 (95% CI: 0.70, 0.92) among DZ twins and 0.92 (95% CI: 0.69, 1.14) for MZ twins. The between-pair estimate in MZ twin pairs (HR: 1.15, 95% CI: 0.87, 1.42) was inconclusive with respect to the size of confounding from familiar shared factors (Fig. 1). The estimates when people aged ≥ 65 years were included, when education as a binary variable was used, and when using the Cox and stratified Cox model, suggested similar result (supplementary material, figure A1-A5).

### Adjustment for BMI and physical fitness

**Fig. 2:**
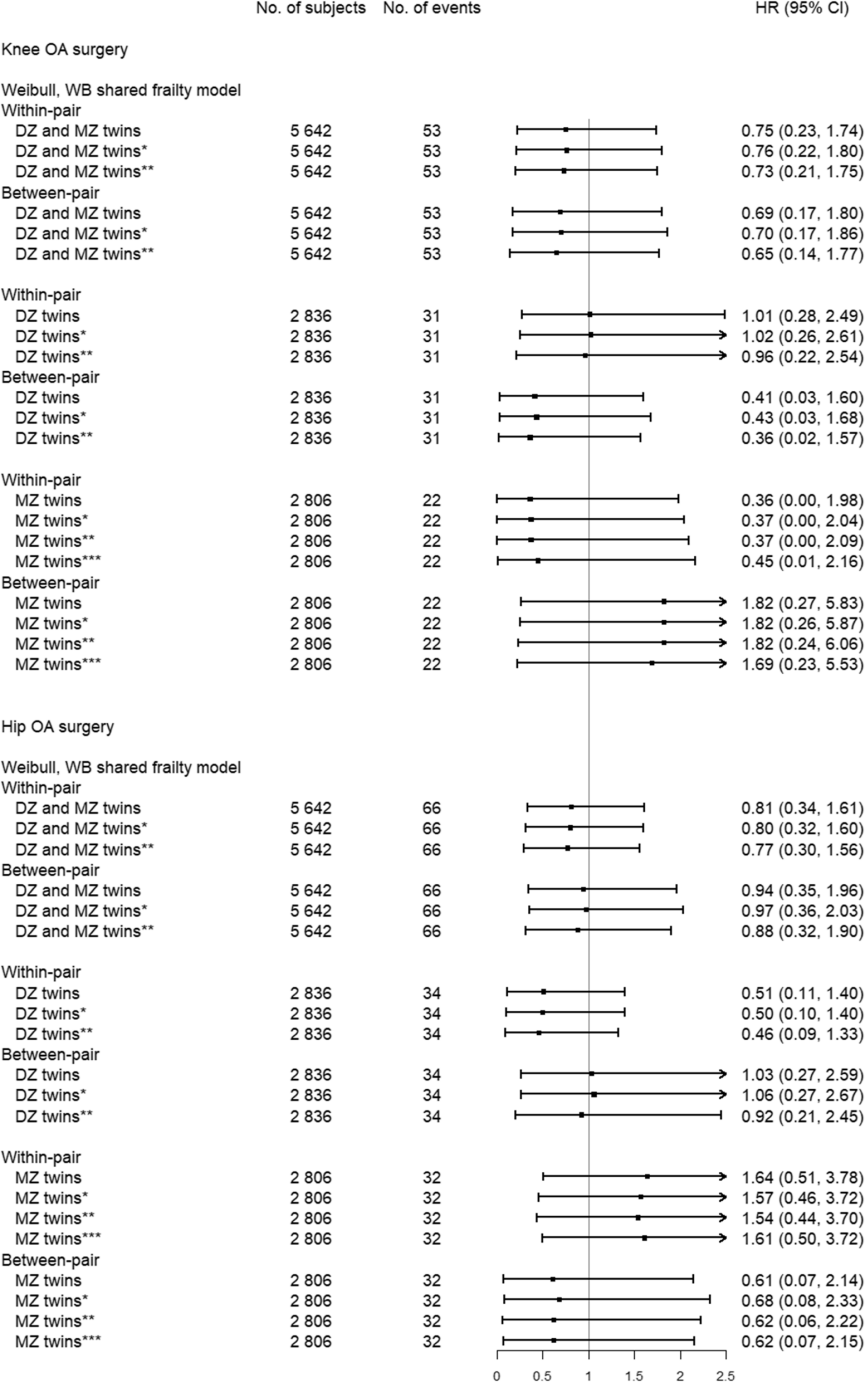
The hazard ratios (HR) per 3 years of education with 95% confidence intervals (CI) for knee and hip OA surgery for male twins born 1951–1980, for matched dizygotic (DZ) and monozygotic (MZ) twins. *Adjusted for body mass index (BMI) at military conscription.**Adjusted for BMI and physical fitness at military conscription. *** Adjusted for BMI, physical fitness at military conscription, birth cohort and place of residence at age 18

When we studied males for whom data on BMI and physical fitness was available (males born 1951–1980)**,** the unadjusted within-twin estimate for knee OA surgery showed a HR of 0.36 (95% CI: 0.00, 1.98) in MZ twins (Fig. 2). This estimate remained similar after adjusting for BMI, physical fitness, and birth cohort and residence at age 18, suggesting no relevant confounding from BMI and physical fitness, however, the CIs were wide precluding us from drawing firm conclusions.

For hip OA surgery, the within-twin estimates for MZ twins remained largely unchanged after adjusting for BMI and physical fitness with point estimates between 1.54–1.64 (Fig. 2).

## Discussion

In this large twin study of 67,071 twins, we have examined the association between educational attainment and knee and hip OA surgery. No association between the individual’s educational attainment and knee OA surgery was found when adjusting for genetics and early-life environment using co-twin control design. Rather, our results suggest that the inverse association between educational attainment and knee OA surgery that is typically seen in observational cohort studies may be confounded by shared familial factors. What concerns hip OA surgery, our results suggest no association between the individual’s educational attainment and the outcome when using co-twin control design. In addition, our results suggest no substantial familial confounding on this association..

### Comparison to previous studies

To our knowledge, this study represents the first attempt at assessing the mechanisms between educational attainment and OA surgery, shedding new and important light on an individual’s possibility to lower the risk of severe OA by reaching a higher education. We found no association between the individual’s educational attainment and OA surgery when adjusting for genetics and early-life environment. Previous studies have reported a protective effect of higher educational attainment on OA outcomes[10–18] and this effect was also seen in our unmatched cohort analyses for knee OA surgery. However, this association was attenuated when examining MZ twins (within-pair estimate of effect on knee OA surgery), indicating that results from our unmatched cohort analyses are due to confounding from genetics and/or early-life environment. Thus, our study emphasises the importance of taking unobserved familial factors including genetics into account when studying the impact of educational attainment on OA, and potentially on other diseases.

### Interpretation of findings

Our result suggests that the protective effect of education on the outcome is confounded by factors shared on a twin-pair or family level such as early-life environment and/or genetics. The finding of familial factors confounding the association between education and OA surgery is in line with the so called life course theory, which demonstrates that adult health is shaped by early childhood events and/or conditions [44]. One could hypothesize that parental characteristics such as parents’ education may have an impact on their child’s health, and in more specific OA surgery. Moreover, it is well-known that parents’ educational attainment is directly associated with their child’s educational attainment [45]. Thus, it might be possible that parental education act as a confounder on the family effect of educational attainment on knee OA surgery found. It has been reported that parents’ educational level is inversely associated with their child being overweight [46] and obesity is an important risk factor for knee OA [33–35]. Life time body weight including birth weight may also be of relevance for OA. For instance, it has been suggested that low birth weight and prematurity is associated with lower educational attainment [47] as well as hip, but not knee, arthroplasty due to OA [48]. When we attempted to address this issue by adjusting the models for BMI and physical fitness measures at age 18 years, typically before the highest educational level is reached, we found that they had little impact on our estimates among males. Future studies should investigate this issue among females.

For knee OA surgery, between-pair analysis showed a tendency of lower HR compared with within- pair analysis, while the opposite was seen for analyses concerning hip OA surgery (Fig. 1). This result might reflect that knee/hip OA surgery is related to individual education (within-pair) differently than the average education in the family (between-pair). This is a known phenomenon in multilevel data (with twin design as a subtype), where the relationship at different levels are not necessarily the same [49].

In this study we did not aim to separate the impact of genetics vs environmental factors on education and OA surgery, for example by using a classical twin design and applying an ACDE model. Such assessment of the amount of familial confounding that is attributed to genetics vs shared environment by comparing DZ and MZ twins may be problematic because of possible violations to the equal environment assumption (EEA), meaning that MZ and DZ twin pairs share their environment to an equal extent. The importance of how big a threat potential violation to the EEA is for drawing inference from the classical twin study is much debated [50–52]. Here, we chose to regard the (probably) more shared environment among MZ twins than among DZ twins as a strength, as it allows for an even better adjustment for shared environmental factors in a co-twin control design.

The finding of a family effect of educational attainment on knee OA surgery but not on hip OA surgery might be related to the more profound role of environmental factors in the progress of knee OA in comparison to hip OA [7, 8, 51]. For example, it is well-known that the genetic contribution is higher for hip OA than for knee OA [6–8], which might explain the differing findings between joint site in our study.

### Strengths and limitations

Important strengths of this study are the inclusion of a population-based sample of twins with limited selection bias and long follow-up time (~ 20 years) and the ability to adjust for all shared confounders applying a matched twin study design to these data. Further, important strengths were that we modelled education level in different ways and that the Swedish healthcare system ensures equal access to procedures such as OA surgeries to all inhabitants. However, there are a couple of important limitations that we would like to highlight. In the present study we did not account for typical individual confounders like knee injuries, occupation etc. We did however, adjust for adult BMI and physical fitness, obtaining similar results. Second, the meaning of educational attainment changes over time, as ex. the university education was rare for persons born in the 40ties as compared to those born in the 80ties, which also may influence our findings.

## Conclusions

By using a twin study design with a large study sample, high-quality data and long follow-up time, we have provided unique insight into the association between educational attainment and the risks of knee and hip OA surgery. Our results suggest that the often reported association between education and knee OA surgery is due to familial confounding. This implies that the previously reported association can be explained by genetics and early-life environment impacting on both educational attainment and knee OA surgery. The same conclusion applies to hip OA surgery, except that there does not seem to be familial confounding on the association.

## Supplementary Information

**Additional file 1.**

## Data Availability

Data were obtained from following registers: the Swedish Twin Registry (STR), the Longitudinal integration database for health insurance and labour market studies (LISA), the Swedish National Patient Register, the Statistics Sweden’s Population Register, the Swedish Military Conscription Registry. The data cannot be made publicly available. According to the General Data Protection Regulation, The Swedish law SFS 2018:218, The Swedish Data Protection Act, the Swedish Ethical Review Act, and the Public Access to Information and Secrecy Act, these types of sensitive data can only be made available after legal review, for researchers who meet the criteria for access to this type of sensitive and confidential data. Interested researchers may contact the following register holders: the Karolinska Institute, Statistics Sweden, the Swedish National Board of Health and Welfare, the Swedish Military Conscription Registry. Computing codes are available in Dahlqwist et.al’s article *Regression standardization and attributable fraction estimation with between-within frailty models for clustered survival data* but also by request to the corresponding author.
