# Supplementary material for: Does lower educational attainment increase the risk of osteoarthritis surgery? a Swedish twin study
Source: BMC Musculoskelet Disord. 2023 Jan 28;24:72. doi: 10.1186/s12891-023-06163-w (PMC9883874; doi:10.1186/s12891-023-06163-w)
Supplement: Supplementary file 1 — Additional file 1. [file 12891_2023_6163_MOESM1_ESM.docx]

**Supplementary material**

Table A1. Descriptive on educational status of the study population when the eldest people were included

| Whole study sample | DZ and MZ complete and incomplete twin pairs | | DZ  complete twin pairs | | MZ  complete twin pairs | |  |
| --- | --- | --- | --- | --- | --- | --- | --- |
|  | Years of education, continuous | | | | | |  |
| Years of education, mean years ± SD | 11.1 ± 2.9 | | 11.0 ± 2.9 | | 11.4 ± 2.9 | |  |
|  | Years of education, categorical | | | | | |  |
|  | <13 | ≥13 | <13 | ≥13 | <13 | ≥13 | Missing |
| Number of people, n | 51,929 | 19,617 | 29,276 | 10,352 | 12,520 | 5,680 | 1,748 |
| Sex,  Females, %  missing | 53.9  0.0 | 55.5  0.0 | 53.0  0.0 | 53.4  0.0 | 55.8  0.0 | 58.4  0.0 | 7.9  84.5 |
| Age at entry,  mean age ± SD | 45.1 ± 11.4 | 40.6 ± 7.5 | 44.9 ± 10.8 | 39.6 ± 7.6 | 44.1 ± 10.9 | 38.4 ± 6.8 | 54.3 ± 15.7 |
| Knee OA surgery, n | 1,828 | 340 | 1,075 | 216 | 427 | 77 | 14 |
| Hip OA surgery, n | 2,051 | 505 | 1,199 | 284 | 470 | 141 | 14 |
| Person-years follow-up, knee OA surgery | 1,213,308 | 389,396 | 714,023 | 223,248 | 287,268 | 104,222 | 9,626 |
| Person-years follow-up, hip OA surgery | 1,210,241 | 387,580 | 712,468 | 222,295 | 286,564 | 103,573 | 9,633 |

Table A2. Descriptive on twin pairs discordant on educational status (continuous variable) when the eldest people were included

|  | DZ and MZ complete twin pairs | | DZ  complete twin pairs | | MZ  complete twin pairs | |
| --- | --- | --- | --- | --- | --- | --- |
|  | Years of education, continuous | | | | | |
| Years of education, mean years ± SD | 11.7 ± 2.8 | | 11.0 ± 2.9 | | 11.4 ± 2.9 | |
|  | Years of education, categorical | | | | | |
|  | <13 | ≥13 | <13 | ≥13 | <13 | ≥13 |
| Number of people, n | 22,696 | 11,848 | 17,252 | 8,464 | 5,444 | 3,384 |
| Females, % | 53.9 | 53.6 | 52.7 | 52.7 | 57.9 | 56.1 |
| Age at entry,  mean age ± SD | 43.5 ± 9.9 | 39.6 ± 7.7 | 43.4 ± 9.8 | 39.9 ± 7.8 | 43.6 ± 10.2 | 39.0 ± 7.3 |
| Knee OA surgery, n | 822 | 235 | 638 | 188 | 184 | 47 |
| Hip OA surgery, n | 926 | 334 | 708 | 247 | 218 | 87 |
| Person-years follow-up, knee OA surgery | 553,676 | 248,596 | 425,739 | 185,368 | 127,937 | 63,228 |
| Person-years follow-up, hip OA surgery | 552,400 | 247,380 | 424,902 | 184,496 | 127,498 | 62,884 |

Table A3. Descriptive on twin pairs discordant on educational status (categorical variable) when the eldest people were included

|  | DZ and MZ complete twin pairs | | DZ  complete twin pairs | | MZ  complete twin pairs | |
| --- | --- | --- | --- | --- | --- | --- |
|  | Years of education, continuous | | | | | |
| Years of education, mean years ± SD | 12.6 ± 2.3 | | 11.0 ± 2.9 | | 11.4 ± 2.9 | |
|  | Years of education, categorical | | | | | |
|  | <13 | ≥13 | <13 | ≥13 | <13 | ≥13 |
| Number of people, n | 5,878 | 5,878 | 4,550 | 4,550 | 1,328 | 1,328 |
| Females, % | 50.6 | 54.3 | 48.5 | 53.3 | 57.6 | 57.6 |
| Age at entry,  mean age ± SD | 40.4 ± 8.2 | 40.4 ± 8.2 | 40.5 ± 8.2 | 40.5 ± 8.2 | 40.2 ± 8.3 | 40.2 ± 8.3 |
| Knee OA surgery, n | 166 | 131 | 136 | 112 | 30 | 19 |
| Hip OA surgery, n | 190 | 184 | 149 | 136 | 41 | 48 |
| Person-years follow-up, knee OA surgery | 132,408 | 134,153 | 104,546 | 105,879 | 27,861 | 28,273 |
| Person-years follow-up, hip OA surgery | 132,233 | 133,493 | 104,468 | 105,461 | 27,765 | 28,032 |

Table A4. Surgery codes used for knee and hip OA surgery

| **Knee OA surgery** | |
| --- | --- |
| 8424 | Bicompartmental knee arthroplasty |
| 8010 | Osteotomy |
| 8428 | Tricompartmental knee arthroplasty |
| 8423 | Unicompartmental knee arthroplasty |
| 8426 | Insertion of patellar prosthesis |
| NGB49 | Primary total prosthetic replacement of knee joint using cement |
| NGB19 | Primary partial prosthetic replacement of knee joint using cement |
| NGK59 | Angulation, rotation or displacement osteotomy of knee or lower leg |
| NGB29 | Primary total prosthetic replacement of knee joint not using cement |
| NGB59 | Primary prosthetic interposition arthroplasty of knee joint |
| NGB53 | Primary patellofemoral prosthesis |
| NGB09 | Primary partial prosthetic replacement of knee joint not using cement |
| NGB39 | Primary total prosthetic replacement of knee joint using hybrid technique |
|  |  |
| **Hip OA surgery** | |
| 8414 | Total prosthetic replacement of hip joint |
| 8010 | Osteotomy |
| 8409 | Other related surgeries (hip arthroplasty without using foreign material) |
| 8419 | Other related surgeries (hip arthroplasty using foreign material) |
| NFB49 | Primary total prosthetic replacement of hip joint using cement |
| NFB29 | Primary total prosthetic replacement of hip joint not using cement |
| NFB39 | Primary total prosthetic replacement of hip joint using hybrid technique |
| NFB99 | Other primary prosthetic replacement of hip joint |
| NFK59 | Angulation, rotation or displacement osteotomy of femur |
| NFB62 | Primary prosthetic replacement of joint surface of femoral head |

The surgery codes are based on the National Board of Health and Welfare´s classification of surgical procedures from 1963 to 1996 ^25^ and 1997 to present ^26^

Table A5. Definition of early-life place of residence

| The counties of Sweden | Categorization in study |
| --- | --- |
| Halland, Blekinge, Östergötland, Jönköping, Kronoberg, Kalmar, Gotland | South of Sweden |
| Södermanland, Uppsala, Västmanland, Örebro, Värmland, Dalarna | Middle of Sweden |
| Gävleborg, Västernorrland, Västerbotten, Jämtland, Norrbotten | North of Sweden |
| Stockholm, Skåne, Västra Götaland | Densely populated county |

Table A6. Definition of years of education

| Statistics Sweden’s  SUN 2000 | Statistics Sweden’s description of the code | Years of education in study |
| --- | --- | --- |
| <200 | Pre-secondary education shorter than 9 years | 7 |
| ≥200 and <300 | Pre-secondary education of at least 9 years | 9 |
| >300 and <320 | Secondary education of at least one semester (but not 2 years) | 10 |
| ≥320 and <330 | Secondary education at least 2 years (but not 3 years) | 11 |
| ≥330 and <400 | Secondary education of 3 years | 12 |
| ≥410 and <500 | Postsecondary education of at least one semester (but not 2 years) | 13 |
| ≥520 and <530 | Postsecondary education of at least 2 years (but not 3 years) | 14 |
| ≥530 and <540 | Postsecondary education of at least 3 years (but not 4 years) | 15 |
| ≥540 and <550 | Postsecondary education of at least 4 years (but not 5 years) | 16 |
| ≥550 and <600 | Postsecondary education of 5 years or longer | 17 |
| ≥600 and <640 | Other / unspecified research education and Licentiate degree | 18 |
| 640 | PhD education | 20 |

Information about Statistics Sweden´s SUN 2000 variable: https://www.scb.se/contentassets/aeeedec0e28c465aa524429407dcd5ba/mis-sun-2000.pdf

Table A7. Descriptive on the educational status for men born 1951-1980 included in the subgroup analysis

|  | DZ and MZ complete and incomplete twin pairs | | DZ  complete twin pairs | | MZ  complete twin pairs | |  |
| --- | --- | --- | --- | --- | --- | --- | --- |
|  | Years of education, continuous | | | | | |  |
| Years of education, mean years ± SD | 12.3 ± 2.3 | | 11.1 ± 2.9 | | 11.5 ± 2.9 | |  |
|  | Years of education, categorical | | | | | |  |
|  | <13 | ≥13 | <13 | ≥13 | <13 | ≥13 |  |
| Number of people, n | 6,883 | 4,100 | 1,796 | 1,040 | 1,666 | 1,140 |  |
| BMI*,  mean kg/m^2^ ± SD | 21.3 ± 2.5  0 | 21.10 ± 2.2 | 21.3 ± 2.4 | 21.1 ± 2.2 | 21.2 ± 2.5 | 21.0 ± 2.1 |  |
| Physical fitness**,  mean Watts ± SD | 264 ± 49 | 292 ± 54 | 260 ± 48 | 290 ± 53 | 266 ± 50 | 294 ± 55 |  |
| Knee OA surgery, n | 73 | 19 | 25 | 6 | 17 | 5 |  |
| Hip OA surgery, n | 78 | 27 | 28 | 6 | 22 | 10 |  |
| Person-years follow-up, knee OA surgery | 123,604 | 63,657 | 35,048 | 17,5814 | 28,798 | 16,140 |  |
| Person-years follow-up, hip OA surgery | 123,612 | 63,598 | 35,090 | 17,559 | 28,763 | 16,123 |  |

*Body mass index at military conscription.
**Physical fitness at military conscription. Measured as maximal aerobic workload in Watts.

Table A8. Descriptive on the educational status for men born 1951-1980 included in the subgroup analysis. The twin pairs are discordant on educational status (continuous variable).

|  | DZ and MZ complete twin pairs | | DZ  complete twin pairs | | MZ  complete twin pairs | |
| --- | --- | --- | --- | --- | --- | --- |
|  | Years of education, continuous | | | | | |
| Years of education, mean years ± SD | 12.8 ± 2.4 | | 11.1 ± 2.9 | | 11.5 ± 2.9 | |
|  | Years of education, categorical | | | | | |
|  | <13 | ≥13 | <13 | ≥13 | <13 | ≥13 |
| Number of people, n | 1,570 | 1,510 | 942 | 818 | 628 | 692 |
| BMI*,  mean kg/m^2^ ± SD | 21.2 ± 2.4 | 21.0 ± 2.1 | 21.3 ± 2.4 | 21.0 ± 2.1 | 21.1 ± 2.4 | 20.9 ± 2.1 |
| Physical fitness**,  mean Watts ± SD | 263 ± 50 | 291 ± 55 | 260 ± 49 | 290 ± 53 | 267 ± 50 | 293 ± 58 |
| Knee OA surgery, n | 14 | 6 | 9 | 4 | 5 | 2 |
| Hip OA surgery, n | 27 | 12 | 21 | 5 | 6 | 7 |
| Person-years follow-up, knee OA surgery | 29,532 | 23,508 | 18,645 | 13,776 | 10,887 | 9,732 |
| Person-years follow-up, hip OA surgery | 29,500 | 23,467 | 18,605 | 13,757 | 10,895 | 9,710 |

*Body mass index at military conscription.
**Physical fitness at military conscription. Measured as maximal aerobic workload in Watt

Table A9. Descriptive on twin pairs discordant on educational status (continuous variable).

| Whole study sample | DZ and MZ complete twin pairs | | DZ  complete twin pairs | | MZ  complete twin pairs | |
| --- | --- | --- | --- | --- | --- | --- |
|  | Years of education, continuous | | | | | |
| Years of education, mean years ± SD | 11.7 ± 2.8 | | 11.1 ± 2.9 | | 11.5 ± 2.9 | |
|  | Years of education, categorical | | | | | |
|  | <13 | ≥13 | <13 | ≥13 | <13 | ≥13 |
| Number of people, n | 21,738 | 11,660 | 16,565 | 8,327 | 5,173 | 3,333 |
| Females, % | 53.5 | 53.6 | 52.2 | 52.6 | 57.3 | 56.1 |
| Age at entry,  mean age ± SD | 42.4 ± 8.5 | 39.1 ± 6.8 | 42.4 ± 8.5 | 39.4 ± 7.0 | 42.3 ± 8.5 | 38.5 ± 6.4 |
| Knee OA surgery, n | 786 | 233 | 612 | 188 | 174 | 45 |
| Hip OA surgery, n | 873 | 323 | 667 | 240 | 206 | 83 |
| Person-years follow-up, knee OA surgery | 534,397 | 244,637 | 412,015 | 182,505 | 122,382 | 62,132 |
| Person-years follow-up, hip OA surgery | 533,363 | 243,533 | 411,421 | 181,717 | 121,942 | 61,816 |

Table A10. Descriptive on twin pairs discordant on educational status (categorical variable).

| Whole study sample | DZ and MZ complete twin pairs | | DZ  complete twin pairs | | MZ  complete twin pairs | |
| --- | --- | --- | --- | --- | --- | --- |
|  | Years of education, continuous | | | | | |
| Years of education, mean years ± SD | 12.6 ± 2.3 | | 11.0 ± 2.9 | | 11.4 ± 2.9 | |
|  | Years of education, categorical | | | | | |
|  | <13 | ≥13 | <13 | ≥13 | <13 | ≥13 |
| Number of people, n | 5,768 | 5,768 | 4,469 | 4,469 | 1,299 | 1,299 |
| Females, % | 50.2 | 54.2 | 48.1 | 53.3 | 57.4 | 57.4 |
| Age at entry,  mean age ± SD | 39.9 ± 7.3 | 39.9 ± 7.3 | 40.0 ± 7.3 | 40.0 ± 7.3 | 39.5 ± 7.2 | 39.5 ± 7.2 |
| Knee OA surgery, n | 158 | 130 | 130 | 112 | 28 | 18 |
| Hip OA surgery, n | 184 | 179 | 144 | 133 | 40 | 46 |
| Person-years follow-up, knee OA surgery | 130,180 | 131,718 | 102,936 | 104,112 | 27,244 | 27,606 |
| Person-years follow-up, hip OA surgery | 129,992 | 131,110 | 102,860 | 103,726 | 27,132 | 27,384 |

Figure A1. The results when the eldest people were included
The hazard ratios (HR) per 3 years of education with 95% confidence intervals (CI) for knee and hip OA surgery, for unmatched and matched dizygotic (DZ) and monozygotic (MZ).


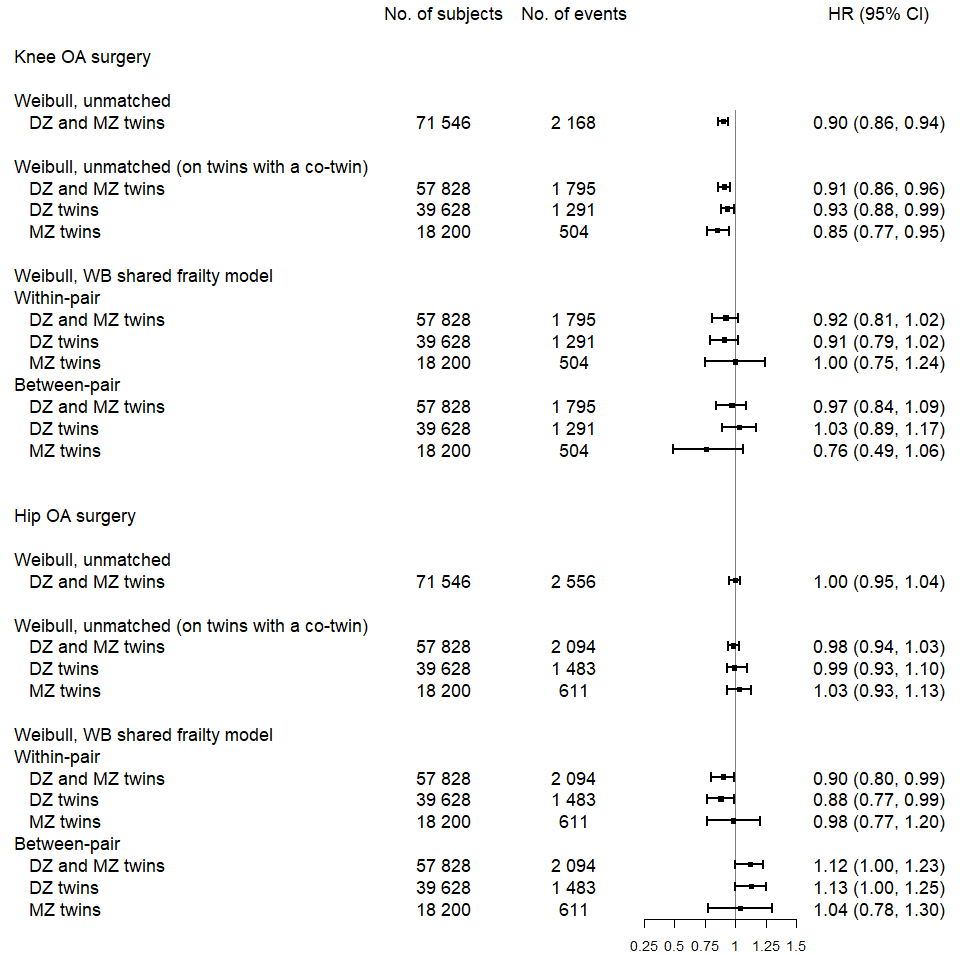


Models were adjusted for sex, birth cohort, and place of residence at age 18.

Figure A2. The hazard ratios (HR) with 95% confidence intervals (CI) for knee and hip OA surgery according to ≥13 years of education with <13 years of education as reference, for unmatched and matched dizygotic (DZ) and monozygotic (MZ) twins.


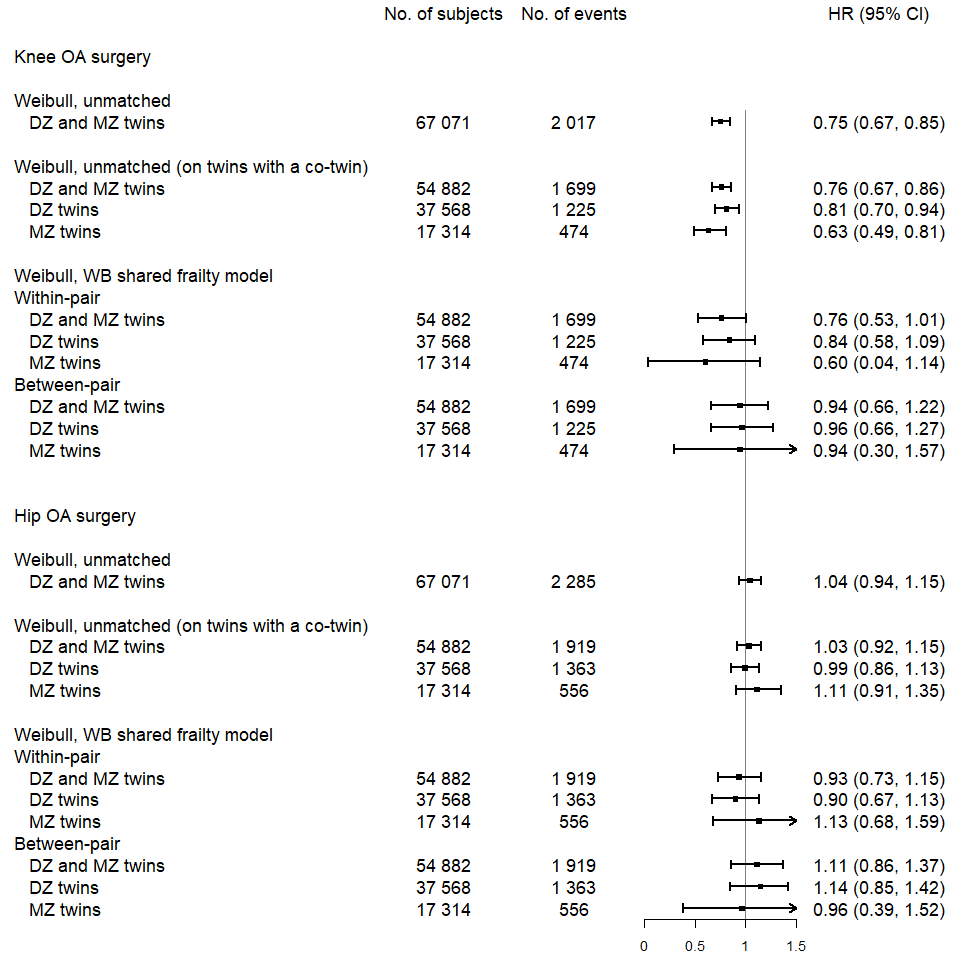


Models were adjusted for sex, birth cohort, and place of residence at age 18.

Figure A3. The results when the eldest people were included
The hazard ratios (HR) with 95% confidence intervals (CI) for knee and hip OA surgery according to ≥13 years of education with <13 years of education as reference, for unmatched and matched dizygotic (DZ) and monozygotic (MZ) twins.


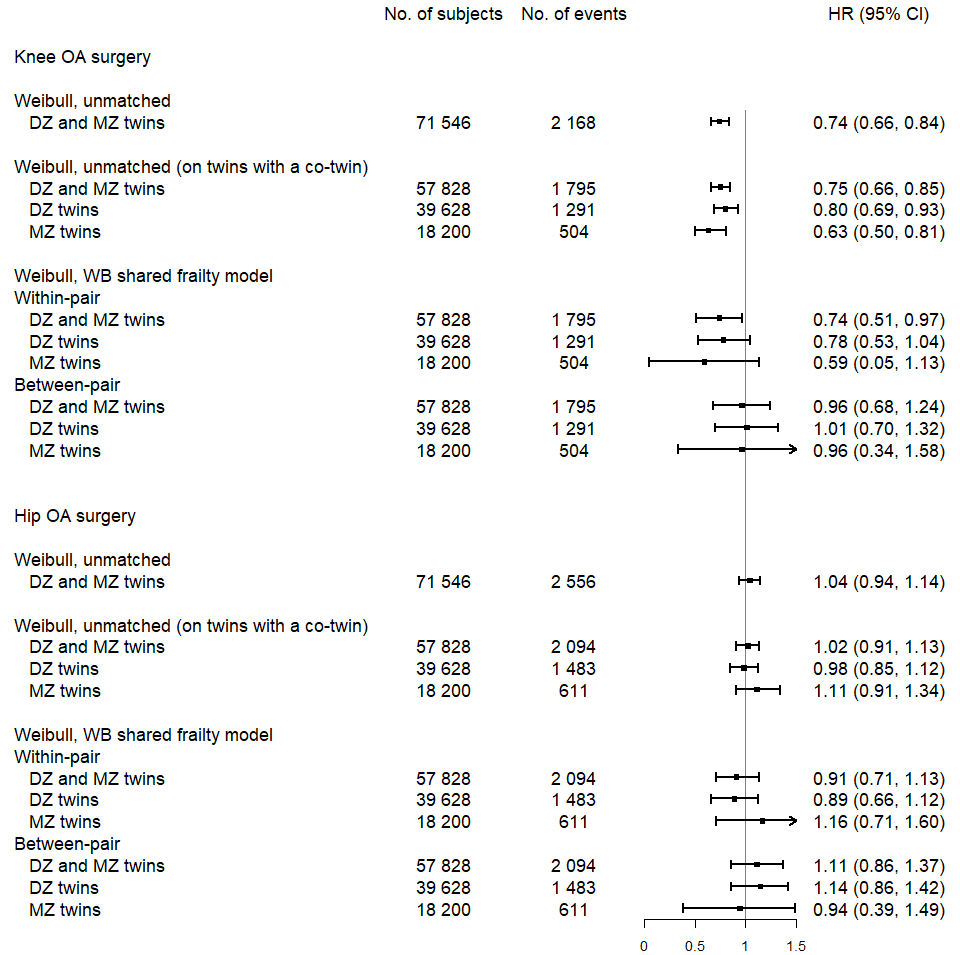


Models were adjusted for sex, birth cohort, and place of residence at age 18.

Figure A4. The hazard ratios (HR) per 3 years of education with 95% confidence intervals (CI) for knee and hip OA surgery, for unmatched and matched dizygotic (DZ) and monozygotic (MZ) twins.


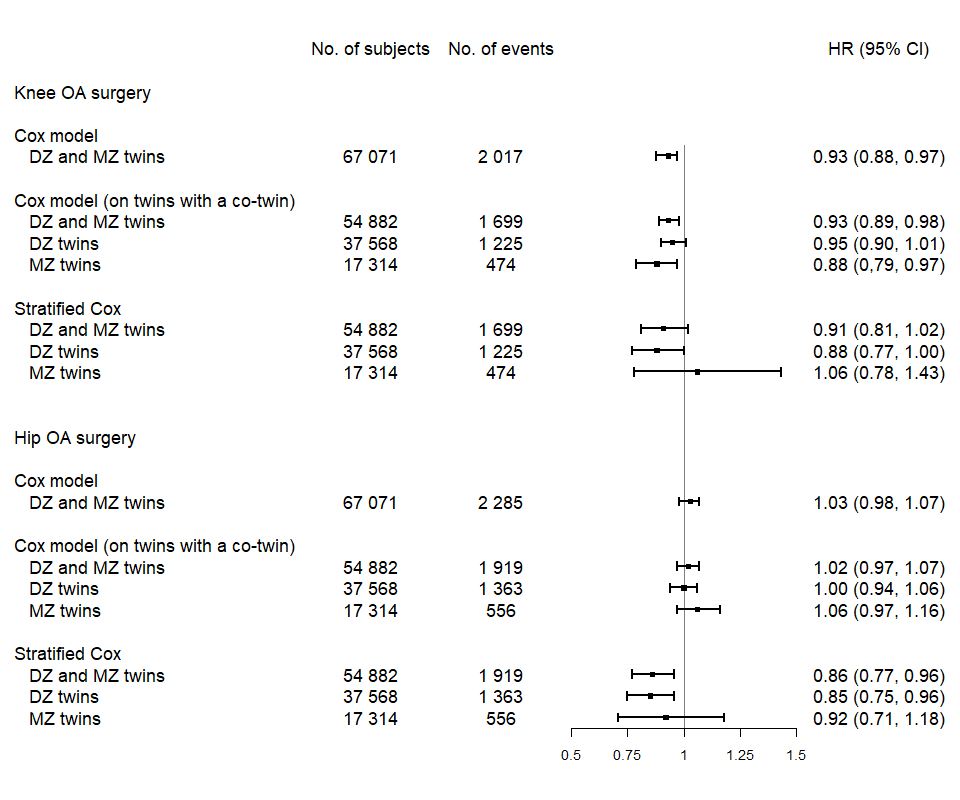


Models were adjusted for sex, birth cohort, and place of residence at age 18.

Figure A5.

The hazard ratios (HR) with 95% confidence intervals (CI) for knee and hip OA surgery according to ≥13 years of education with <13 years of education as reference, for unmatched and matched dizygotic (DZ) and monozygotic (MZ) twins.


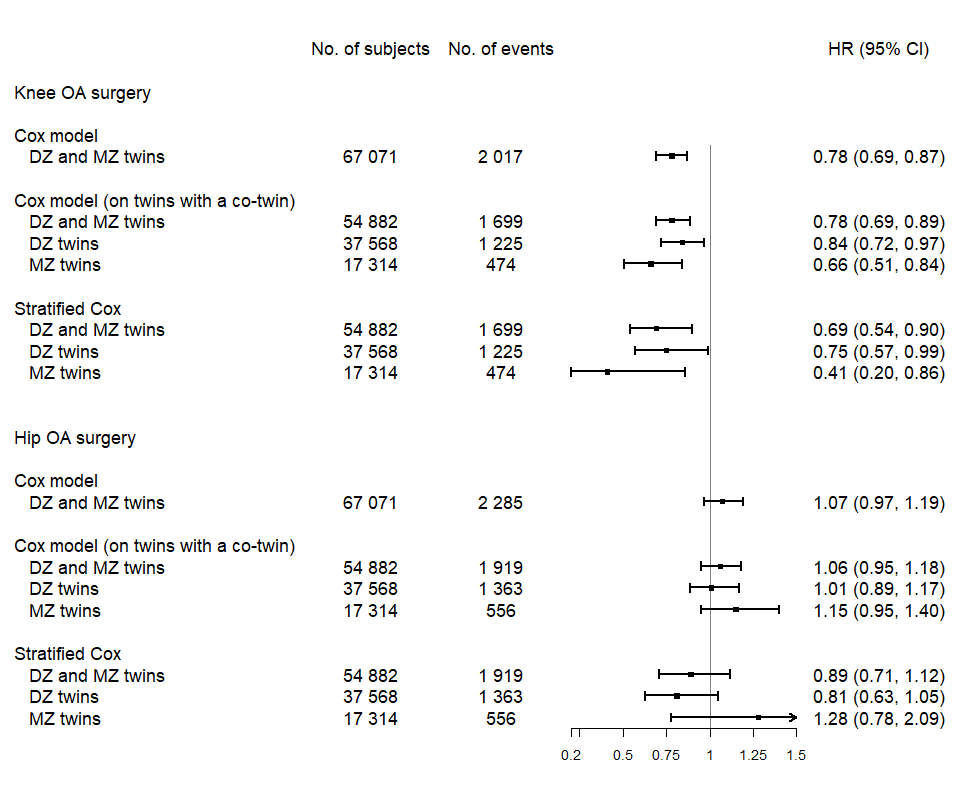


Models were adjusted for sex, birth cohort, and place of residence at age 18.
